# Different extraction methods of biologically active components from propolis: a preliminary study

**DOI:** 10.1186/1752-153X-1-13

**Published:** 2007-06-07

**Authors:** Boryana Trusheva, Dorina Trunkova, Vassya Bankova

**Affiliations:** 1 Institute of Organic Chemistry with the Centre of Phytochemistry, Bulgarian Academy of Sciences, Acad. G. Bonchev str., bl. 9, 1113 Sofia, Bulgaria

## Abstract

**Background:**

Propolis is widely used in apitherapy, preparations, and food and beverage additives. Various extraction techniques were applied in the extraction of the biologically active constituents of poplar type propolis in order to compare their efficiency. The methods employed were: traditional maceration extraction, ultrasound extraction (UE), and microwave assisted extraction (MAE).

**Results:**

The total amounts of extracted phenolics and flavonoids were determined, and the effectiveness of the methods compared. MAE was very rapid but led to the extraction of a large amount of non-phenolic and non-flavonoid material. UE gave the highest percentage of extracted phenolics.

**Conclusion:**

Compared to the maceration extraction, MAE and UE methods provided high extraction yield, requiring short timeframes and less labour. UE was shown to be the most efficient method based on yield, extraction time and selectivity.

## Background

The use of propolis as a remedy has a long history [1]. In addition, preparations, as well as food and beverage additives containing propolis extracts can be found on the market in numerous countries [2]. It is generally accepted that the solvent of choice for the extraction of biologically active components of propolis (mainly phenolics, including different types of flavonoids) is 70% ethanol, with most commercial products extracted using this solvent system [3,4]. The traditional method (maceration), however, is time consuming, requiring timeframes from 2 to 10 days [4,5]. Recently, modern extraction methods have been developed for the fast and efficient extraction of organic compounds from solid matrices, with microwave assisted extraction (MAE) and ultrasonic extraction (UE) among the most promising for the extraction of natural products [6,7]. MAE is the process of using microwave energy to heat solvents in contact with a sample in order to partition some chemical components from the matrix into the solvent. The benefits of UE are thought to be due mainly to the mechanic effects of acoustic cavitation. Both methods have recently demonstrated the potential to reduce extraction times significantly and increase extraction yields in a number of studies on medicinal plants [8]. The aim of this preliminary study is to compare the effectiveness of the extraction of bioactive components from poplar propolis (phenolics, flavones/flavonols and flavanones/dihydroflavonols [9] using the maceration method, MAE and UE. We have chosen to quantify groups of active compounds with the same or similar chemical structure rather than individual substances. Recently, it has been demonstrated that the concentration of such compound groups in propolis extracts correlates much better with the levels of antibacterial activity and is more informative than the concentration of individual components [9,10]. We believe that this study is the first application of MAE to propolis.

## Results and discussion

Three extraction methods were employed in order to obtain the biologically active components of poplar type (European) propolis. The results are summarized in Table 1.

**Table 1:** Comparison of the extraction yields (percentage of extracted matter from raw propolis) for the maceration extraction, UE and MAE*

Extraction type	Propolis/solvent (*m*/V)	Time	Total phenolics, %	Total flavones/flavonols, %	Total flavanones/dihydroflavonols, %	Total extract, %
Maceration	1:20	72 h	44 ± 2	8.6 ± 0.1	18 ± 1	58 ± 3
Maceration	1:10	72 h	43 ± 2	8.8 ± 0.1	19 ± 2	55 ± 1
UE	1:20	10 min	35.9 ± 0.5	9.4 ± 0.2	20 ± 5	41 ± 1
UE	1:20	30 min	50 ± 1	8.6 ± 0.1	23 ± 2	50 ± 1
UE	1:10	30 min	52 ± 3^a,b,c^	9.6 ± 0.8^a^	22 ± 2^a^	53 ± 3^a,b,c^
MAE	1:20	2 × 10 s	40 ± 1	9.3 ± 0.1	23 ± 3	73 ± 2
MAE	1:10	2 × 10 s	40.4 ± 0.6^a,b,c^	8.7 ± 0.1^a^	20 ± 1^a^	75 ± 0^a,b,c^
MAE	1:10	3 × 10 s	24.4 ± 0.4	10.7 ± 1.7	7.5 ± 0.6	66 ± 5

• Mean values of three extraction ± SD;

• ^a ^values compared with those obtained via maceration, (propolis/solvent 1:10),

• ^b ^values significantly different from those obtained by maceration (propolis/solvent 1:10), p < 0.05 (Student's t-test),

• ^c ^values compared and found significantly different, p < 0.05 (Student's t-test)

In all three extraction techniques the amount of solvent used (10:1 or 20:1) does not significantly influence the extraction yield (ANOVA). This is an important finding because it demonstrates that the use of solvent/propolis ratios larger than 10:1 (v/w) is unnecessary, leading only to solvent and energy losses.

In the case of ultrasound extraction, the yield of biologically active constituents increases with the time. It is clear that a 30 min sonication should be sufficient to extract the available amount of phenolics and flavonoids. In general, this confirms the results of a previous study [12].

With MAE the situation was different. Surprisingly, an additional cycle of irradiation (3 × 10 s instead of 2 × 10 s) resulted in a drastic decrease in the yield of phenolics and flavanones/dihydroflavonols. The small increase in percentage of the extracted flavones/flavonols was not statistically significant. Presumably, the high energy influx in this case leads to chemical changes (most probably oxidation) of the phenolic compounds (including flavonoids) and to lower amounts of these substances detected in the extracts.

The three extraction methods differed mainly in the total percentage of phenolics extracted, with UE giving the highest yield. UE led also to the extraction of the highest amount of flavanones and dihydroflavonols, while in the total extraction of flavones/flavonols, UE and MAE were did not show statistically significant differences. The results demonstrate that the use of ultrasound and of microwave extractions greatly reduce the extraction time.

The percentage of total extract obtained with a 70% ethanol solvent system also varies significantly with the extraction method, much more so than the amount of extracted active compounds. This is important, because the greater percentage of total extract and similar percentage of extracted active compounds means that larger amounts of waxes have been extracted. This was especially the case with MAE where the extracted phenolics constituted 40% of the mass of the raw propolis while the total extract was 70% of the raw propolis. UE resulted almost only in the extraction of active components and minimum wax.

## Conclusion

Compared to the maceration extraction, MAE and UE methods provided high extraction yield, requiring short timeframes and less labour

MAE of bioactive phenolics and flavonoids from poplar type propolis was found to be a very fast extraction method, compared to maceration and even UE. However, the extraction selectivity was low, with significant amounts of unwanted wax was found to have been extracted. In addition, longer irradiation times resulted in a decrease in the percentage of extracted active components, presumably owing to degradation processes.

UE has been shown to be the most efficient extraction method, taking into consideration yield, short extraction time (30 min) and high selectivity.

## Experimental

### Propolis

The propolis sample was obtained from the National Institute of Apiculture in Bologna, Italy, and its poplar origin proved using TLC test [11]. The whole sample (51 g) was frozen, ground and homogenized prior to beginning extraction experiments.

### Extraction experiments

2 g of propolis were used in each of the extraction experiments. The extraction solvent was 70% ethanol. After extraction the sample was filtered and the filtrate diluted to 100 mL with 70% ethanol in a volumetric flask. Analysis then followed according to the methods described later. Each extraction experiment was performed in triplicate.

### Maceration extraction

For maceration extraction, the material was placed in an Erlenmayer flask, the corresponding amount of solvent was added (details in Table 1) with the sample left to macerate in the dark for 72 h at room temperature.

### Ultrasound extraction (UE)

The UE was carried out using a 300 W ultrasonic bath. The sample was placed in an Erlenmayer flask with the corresponding amount of solvent and was treated with ultrasound at 25°C for a given duration (Table 1).

### Microwave assisted extraction (MAE)

MAE was performed using a multimodal household microwave oven Panasonic NN-S255W at 800 W and a 100 mL flask exposed to microwave irradiation (irradiation cycle: 10 s power on, followed by 10 s power off) for a given duration (Table 1).

### Analyses of the biologically active compound

The analysis was performed as described in a previous study [9]. In brief, it consisted of the following:

### Total amount of extract

2 mL of each extract were evaporated *in vacuo *to dryness and a constant weight. The percentage of dry extracts was calculated and the mean of the three replicates was determined.

### Total flavone and flavonol content

An aliquot (2 mL) of each extract, 20 mL methanol and 1 mL 5% AlCl_3 _in methanol (*m*/V) were mixed in a volumetric flask and the volume made up to 50 mL with methanol. The mixture was left for 30 min and the absorbance at 425 nm was measured. Calibration was performed using galangin as reference compound. All the values found were within the linear range of the methods, recovery 102%. For each extract, three measurements were performed.

### Total flavanone and dihydroflavonol content

An aliquot (1 mL) of each extract and 2 mL of DNP (2,4-dinitrophenylhydrazine) solution (1 g DNP in 2 mL 96% sulfuric acid, diluted to 100 mL with methanol in a volumetric flask) were heated at 50°C for 50 min. After cooling to room temperature, the mixture was diluted to 10 mL with 10% KOH in methanol (*m*/V). 1 mL of the resulting solution was added to 10 mL methanol and was diluted to 50 mL with methanol (volumetric flasks). Absorbance was measured at 486 nm. Calibration was performed using pinocembrin as reference compound. All the values found were within the linear range of the method, recovery .108%. For each extract, three measurements were performed.

### Total phenolics content

An aliquot (1 mL) of each extract was transferred to a 50 mL volumetric flask, containing 15 mL distilled water. To this were added 4 mL of the Folin-Ciocalteu reagent and 6 mL of a 20% sodium carbonate solution (*m*/V). The volume was made up with distilled water to 50 mL. The sample was left for 2 h and the absorbance at 760 nm was measured. Calibration was performed using a 2:1 (w/w) reference mixture of pinocembrin and galangin as a standard. All the values found were within the linear range of the method, recovery .90% [using other calibration standards, e.g. gallic acid or caffeic acid, the recovery was much lower [9]]. For each extract, three measurements were performed.

## Authors' contributions

BT performed the extractions and participated in the chemical analyses. DT participated in the chemical analyses. VB conceived of the study, participated in its design and coordination and performed the statistical analysis. All authors read and approved the final manuscript.
